# In-depth insight into the short-term effect of floor management practice on young apple trees development and soil microbial biodiversity and activity

**DOI:** 10.1371/journal.pone.0329979

**Published:** 2025-08-07

**Authors:** Ewa M. Furmanczyk, Eligio Malusà

**Affiliations:** Department of Plant Protection, The National Institute of Horticultural Research, Skierniewice, Poland; Canakkale Onsekiz Mart University, TÜRKIYE

## Abstract

Living mulches can be considered a practice providing multi-level benefits and several ecosystem services in orchards. Two multifunctional living mulches (Mix1 – *Trifolium repens* (20%) and *Festuca ovina* (80%), Mix2 – a mixture of 10 different species suitable as flowering strip) sown during the establishment of an apple orchard, were evaluated in relation to the impact on the soil nutrient content and bacterial microbiome, as well as the growth and yield potential of the apple trees. Notwithstanding the increase of N-nitrate and N-ammonia forms induced in the soil, both living mulches mixtures limited the growth and yield potential of the apple trees during the first two growing seasons. The two mixtures induced divergent effects on the biodiversity, activity and metabolic potential of the soil bacterial microbiome, not always increasing them compared to natural cover (control). Such effects could be related to modifications that both mixtures induced in the bacterial community capacity of metabolizing some classes of C-sources compared to control. This was particularly evident for Mix2 and in summer, when all classes of C-sources utilised by the bacterial community for its metabolism resulted to be significantly more exploited than in other seasons. In conclusion, the impact of very diverse living mulch mixtures on the growth, yield potential and overall nutrient status of the young apple trees could be related to modification of the tree physiological status, indirectly derived from the modifications observed on the soil bacterial communities’ composition and metabolic capacity during the whole vegetative season. These results could provide hints for the correct soil management of the orchard to foster the exploitation of soil microbiome suitable for the apple trees development and production.

## Introduction

Introduction of additional plant species or crops into an orchard within the tree row can be considered a practice that enhances the biodiversity of the entire orchard, not only by introducing new plant species, but also by recruiting pollinating insects or sheltering beneficial insects that could positively affect the fruit yield and can help reduce pests populations [1,2]. However, the introduction of additional plants can provide multi-level benefits. Legume plants, for example, could also play an additional role in the agroecosystem by supplying nitrogen to the soil through symbiosis with rhizobia strains [3]. Using herbs as living mulches can also provide an additional source of income for the farmers [4,5]. Flowering species are generally used to establish flower strips on the perimeter of the orchards to provide various ecosystem services [6–9], but have seldom been exploited as living mulches, i.e., introducing them “within” the orchard. Nevertheless, when designing an agroecological orchard, it is important to consider that accompanying plants use the same environmental resources as the main crop, e.g., soil nutrients or sun light, and that the tree needs differ depending on the orchard age. Past studies on living mulches based on simple mixtures of grasses and leguminous species have generally evaluated their impact on weeds control or tree performance [10–12], and to a limited extent on the soil biological features [13].

Soil microbiome plays a crucial role in maintaining soil health and balance [14]. Microbial communities are involved in nutrient cycling, carbon sequestration, plant growth promotion and many other processes [15]. Agricultural management practices, including fertilizer application, tillage, or cover crops, influence the soil microbial communities composition and function [16]. The majority of studies on the impact of cover crops on the soil microbiome are concerned with annual cropping systems, where cover crops are present in the field during fallow periods [17]. Few works on perennial crops, including orchards or vineyards, have analysed the impact of living mulches on biodiversity and microorganisms abundance during the growing season, as well as on soil enzymatic activity: in general showing their increase after applying such soil management practices [13,18–20]. Nevertheless, there is the need to better understand how these practices are affecting the soil microbiome dynamics, its functions and potential provision of ecosystem services, due to the high complexity of its relationships and interactions with the plant and the environmental conditions [21–23]. Such knowledge could fill some research gaps about the interaction of living mulches with the tree crop in relation also to the plant-soil-microbiome relations and support designing a cropping system more resilient toward abiotic and biotic stresses, reducing the need of external inputs, assuring at the same time the farmer economic expectations. Such knowledge would be of utmost importance particularly for apple cropping system, one of the most economically important fruit crops in the world and in Poland, where it accounts for about 150.000 ha, delivering about 4M tons of fruits yearly, contributing roughly 30% of the total EU apple harvest [24].

The present study investigated two very diverse plant mixtures designed for the establishment of multifunctional living mulches suitable, to a different extent, for increasing soil nutrients, reduce weeds competition, favouring pollinators and beneficial arthropods. The working hypothesis was to verify whether the increase of above-ground biodiversity, considering the plants coenosis, could improve the soil microbial biodiversity and functions as well, thus providing additional ecosystem services to the cropping system. Therefore, the living mulches effect on the soil nutrient content and bacterial microbiome, as well as on the development of the apple trees was assessed in the first years of orchard establishment.

## Materials and methods

### Experimental site and management practices

The experiment was conducted in a commercial apple orchard located in Leczeszyce (51°46’18” N, 20°47’49” E, central Poland). The location is characterized by the average annual temperature of 10°C and an average annual precipitation of 650 mm. The orchard was established on 28^th^ April 2021 by planting 2-year old saplings cv. Golden Delicious on M9 rootstock, spaced at 3.0 m x 0.9 m (3700 trees/ha). Plant protection was carried out according to the integrated production guidelines [25,26], according to the needs. Two plant mixtures were tested as living mulches: one consisting of *Trifolium repens* (20%) and *Festuca ovina* (80%) and second being a mixture of 10 different species designed for flowering strips establishment composed of: *Trifolium repens* (10%), *Festuca ovina* (80%), *Plantago lanceolata* (0.5%), *Sanguisorba minor* (4%), *Silene vulgaris* (1.5%), *Achillea millefolium* (1.0%), *Dianthus deltoides* (1.0%), *Leontodon hispidus* (0.5%), *Thymus pulegioides* (0.5%) and *Prunella vulgaris* (1.0%). These mixtures were commercially available and recommended for either covering the soil to reduce erosion and weeds control (Mix1) or to support pollinators and beneficial arthropods as flowering strip (Mix2) consisting of perennial species commonly found in Poland and Europe. The plant mixtures were sown along the tree row on 26^th^ May 2021, four weeks after trees planting (10 g/m^2^). The control was set as natural cover with herbicide treatment during the season: two hand weeding during first season and two chemical treatments with Gallup 360 SL (glyphosate 360 g/l) and Kileo 400 SL (240 g/l glyphosate and 160 g/l 2,4-dichlorophenoxyacetic acid) during second season, on 28^th^ May and 26^th^ July 2022 respectively. Each treatment consisted of three replicates (5 trees each). The field research was carried out in a private orchard. No animals or whole plants were collected from the environment. The study involved only soil and leaf sampling, which did not require any official permits.

### Plant growth and yield potential assessment

Plant growth was assessed by determining the increase of the average trunk growth based on two perpendicular measurements performed about 20 cm above the grafting union, and counting the number of one-year-old shoots and their length at the end of each vegetative period (BBCH 95) on each tree of the replicates (30^th^ Sep 2021, 15^th^ Nov 2022 respectively).

The blooming intensity and fruitlets number were considered as proxy of the yield potential. The number of flowers and fruitlets was counted per each tree during the second vegetative season (on 12^th^ May – BBCH 65 and 11^th^ July 2022 – BBCH 72, respectively). A hail event made not possible to estimate the fruit yield in that season.

### Leaf nutrient analysis

Leaf samples were collected randomly from each replicate in July 2022, gathering 60 mature leaves from current-year shoots from the central section of the tree. The leaves were washed with distilled water, dried at the temperature of 60°C in a forced-air oven, then ground in a Wiley stainless-steel mill. The samples were microwave digested in HNO_3_, using closed Teflon vessels. Inductively coupled plasma spectrometry (ICP Model OPTIMA 2000DV, Perkin Elmer, USA) was used to determine P, K, Na, Ca, and Mg [27]. The N content was determined using the Kjeldahl method (Vapodest, Gerhardt, Germany) after mineralization in concentrated sulfuric acid in the presence of copper-potassium catalyst [27].

### Soil sample collection

Soil samples for chemical and biological analyses were collected three times during the 2022 growing season within well-established living mulches (12^th^ May, 11^th^ July and 14^th^ September). Sampling was performed using Egner’s auger (2.5 cm diameter) collecting at least 10 subsamples at 0–20 cm depth in the volume of soil near the tree root system (30 cm around the trunk). The subsamples were pooled and mixed to form the analytical sample. Visible animals, organic parts (plant residues, roots) as well as small stones were removed at this stage. The samples were stored in 4°C for up to one week or proceeded immediately for soil chemical analysis, and microbial biodiversity and activity determination.

### Soil chemical analysis

The soil samples were analyzed in terms of pH, salinity, and the levels of macronutrients: N-NO_3_, N-NH_4_, P, K, Ca and Mg. After drying at a temp. of 65°C, the soil was mineralized in concentrated nitric acid in a Candela Mars 5 microwave digestion oven. Nitrogen was determined colorimetrically with a Skalar SanPlus automated flow analyzer [28], and phosphorus and potassium were analyzed with a Perkin Elmer OPTIMA 2000 DV plasma spectrometer [29]. The pH of the soil samples was determined on KCl extract: 10 g of homogenized and air-dried soil was mixed with 25 mL of 1 M KCl, and the measurement was carried out on the solution after 24 h using a pH meter (Fisher Scientific, Warsaw, Poland).

### Soil microbial biodiversity and activity assessment

Soil microbial biodiversity and activity were determined using the BIOLOG system and EcoPlates plates. A suspension was obtained from 1 g of soil using sterile distilled water (1:9 w:v) and serially diluted three times. Plates were inoculated with 100 µl of appropriate soil suspension (10^−3^) on each well and then incubated in the dark at 26°C. After 72h the absorbance at 590 nm (OD) was determined.

The activity of microorganisms was estimated on the basis of Average Well Colour Development (AWCD) [30] and the overall activity index or activity index for each compound class were calculated according to the formula:


$$AWCD=\sum {OD}_{i}/n$$ (1)

where OD_i_ is the optical density of the individual wells and n is the number of substrates included in specific category (31 for overall AWCD, 10 for carbohydrates, 7 for carboxylic acids, 6 for amino acids, 4 for polymers, 2 for phenolic compounds and 2 for amines). The microbial biodiversity index was estimated using the Shannon-Weaver coefficient (H):


$$H=-\sum p_{i}(ln{\;p}_{i})$$ (2)

where p_i_ is the level of microbial activity in each well (ODi) divided by the activity in all the wells (Ʃ OD_i_) [31]. When calculating the level of activity of microorganisms for the H index and the amount of metabolized substrates, the threshold value OD = OD_i_ – OD_(control well)_ was used. Substrate richness (S), which is the number of utilized carbon substrates, was calculated using an OD590 value of 0.500 as threshold for positive response. The results were used for comparison and visualization of the potential metabolic profiles of the soil microbial communities.

### Statistical analysis

Statistical analysis of data was performed using the R software version 4.1.3 [32]. The Shapiro-Wilk test was used to verify if the data followed a normal distribution and the Levene’s test was used to verify the homogeneity of variances. The data were thus analysed by ANOVA and means differences tested with Tukey’s test at p ≤ 0.05 with HSD test function from the “agricolae” package. In case of not normal distribution, the non-parametric Kruskal-Wallis analysis with Fisher’s least significant difference post hoc test was utilized, introducing the Benjamini-Hochberg correction, with significance set at p ≤ 0.05, using the Kruskal function from the “agricolae” package. PCA was performed using “prcomp” command from the “stats” package and visualized using “ggplot2” and “ggfortify” packages. Heatmaps were generated using “heatmap.2” command from “gplots” package.

## Results

### Plant growth response

Both tested living mulches mixtures partly negatively affected the main crop growth during the first two growing seasons (Table 1). Indeed, apple trees showed lower trunk diameter growth after the first season particularly when associated with *Trifolium repens* and *Festuca ovina* living mulch mixture than control trees. Even though no effect on trunk growth was observed during the second season, the control trees showed significantly higher trunk growth in 2-year perspective than the two mixtures. Both tested living mulch mixtures did not affect the number of 1-year-old shoots during both seasons, but reduced significantly their length over the 2 years, particularly Mix 2 (Table 1). As a consequence, after 2 vegetative seasons control trees developed a significantly higher total length of 1-year shoots (644.9 cm), compared to Mix1 (505.9 cm) and Mix2 (422.5 cm). The flowering intensity and yield potential were also affected by the living mulch mixtures (Table 1). The number of flowers per tree resulted was about 60% and 75% of the value observed for control trees, respectively for Mix 1 and Mix 2, which was reflected in a similar lower rate of fruitlets.

**Table 1:** Apple tree growth and potential yield as affected by two living mulch mixtures.

Season	Control	Mix1	Mix2
Trunk diameter growth (mm)			
2021	2.6 ± 3.4 a	0.5 ± 1.6 b	1.3 ± 1.9 ab
2022	3.8 ± 3.4 a	3.6 ± 1.5 a	3.2 ± 2.6 a
Cumulative	6.4 ± 1.7 a	4.1 ± 1.3 b	4.5 ± 1.7 b
One-Year Shoot Number			
2021	10.9 ± 2.2 a	10.3 ± 2.4 a	9.8 ± 2.1 a
2022	29.7 ± 5 a	28.1 ± 4.8 a	25.9 ± 6.8 a
Cumulative	40.6 ± 5.4 a	38.3 ± 5.4 a	35.7 ± 6.1 a
Average Length of One-Year Shoot (cm)			
2021	12.6 ± 3.0 a	10.4 ± 3.1 a	11.8 ± 2.7 a
2022	17.1 ± 3.1 a	14.0 ± 3.4 ab	11.0 ± 4.2 b
Cumulative	15.8 ± 2.7 a	12.9 ± 2.9 b	11.5 ± 3.1 b
Total Length of One-Year Shoot (cm)			
2021	135.9 ± 31.3 a	104.0 ± 31.9 a	115.2 ± 39.8 a
2022	509.0 ± 143.7 a	401.9 ± 152.4 b	307.3 ± 188.7 c
Cumulative	644.9 ± 158.9 a	505.9 ± 171.9 b	422.5 ± 177.0 b
Flowering intensity (flowers number)			
2022	194.1 ± 55.9 a	114.3 ± 63.8 b	143.9 ± 35.4 ab
Fruiting potential (fruitlets number)			
2022	28.7 ± 7.3 a	21.1 ± 11.3 ab	19.9 ± 7.8 b

Mean±SD, n = 15. Letters show statistically significant differences for the comparison of data in the row for p ≤ 0.05.

### Nutrient cycling in soil and trees

**Fig 1:**
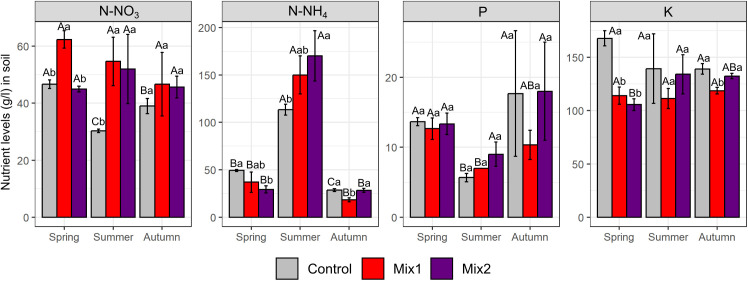
Dynamic and concentration of soil nutrients as affected by the introduction of two kinds of living mulches. Mean±SD, n = 3. Letters show statistically significant differences for p ≤ 0.05 (A-C for the same treatment in different timepoints, a-b for different treatments in the same timepoint).

The two mixtures affected both soil nutrients content and their seasons dynamics, as well as the soil chemical buffering capacity (Fig 1 and S1 Table). The presence of *T. repense*, a leguminous, in Mix1 induced a higher N-NO_3_ content in the soil, particularly compared to the control, through the spring and summer sampling points, and avoiding the decrease occurring during summer observed in the control. Even though the seasonal dynamics of N-NH_4_ was only slightly affected by the mixtures, being in general similar to the control (any potential differences arise only from observed deviations between replicates), a higher content was determined in the summer samples from Mix2 treatment. Considering the other nutrients, both mixtures only marginally affected their dynamics, changes occurring only in case of Mix2 for K and P. Considering the level of the other nutrients both mixtures induced a reduction of K content, but not always significant, while no differences were observed for the other cations and P contents. Both mixtures stabilized the pH during the season compared to control, while no major changes occurred to the salinity level (S1 Table).

The two kinds of living mulches did not affect N content in leaves, but Mix1 statistically reduced K and P content, while increasing Mg content compared to control and Mix2, which only induced a reduction in Ca content in the leaves (Table 2).

**Table 2:** Macroelements content in leaves (% of dry weight) as affected by two kinds of living mulches.

Treatment	N	P	K	Ca	Mg
Control	2.36 a	0.18 a	1.80 a	1.66 a	0.27 b
Mix1	2.27 a	0.16 b	1.48 b	1.75 a	0.33 a
Mix2	2.32 a	0.18 a	1.80 a	1.42 b	0.25 b

Different letters show statistically significant differences for p ≤ 0.05.

**Fig 2:**
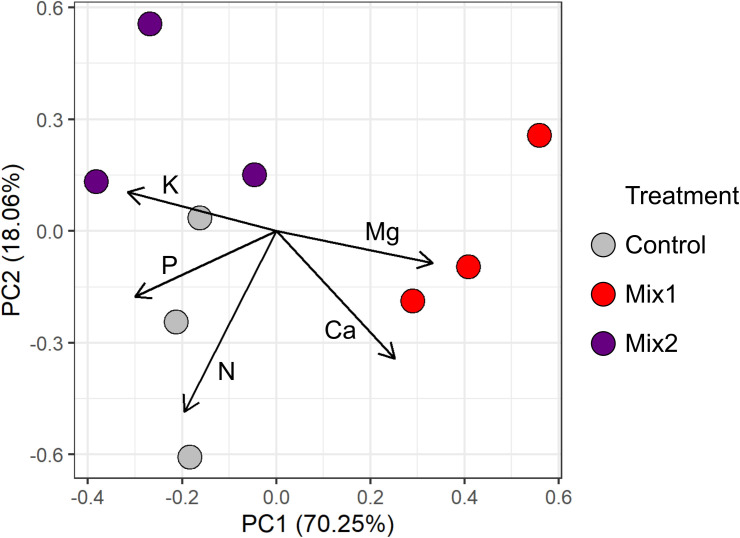
PCA based on the leaves total nutrients content. Each point represents a replicate for the specific treatment.

However, the analysis of all macroelements levels in leaves discriminated both Mix1 and Mix2 treatments from the control and among them (Fig 2).

### Living mulches impact on soil microbiome

The comparison of microbial activity level (AWCD), biodiversity Index (H’) and substrate richness (S) of the soil samples collected across the 2022 vegetative season are presented in Table 3.

**Table 3:** The impact of two kinds of living mulches on soil bacterial activity and biodiversity indices.

Treatment	AWCD			H			S		
	Spring	Summer	Autumn	Spring	Summer	Autumn	Spring	Summer	Autumn
Control	0.93 a	1.53 b	1.11 a	2.98 b	3.31 a	2.93 b	17.67 b	29.33 a	17.67 b
Mix1	1.00 a	1.55 b	0.82 b	3.08 a	3.27 a	2.87 b	20.67 a	27.67 a	16.33 b
Mix2	0.75 b	1.73 a	1.07 a	2.86 c	3.32 a	3.12 a	15.00 b	30.00 a	22.00 a
Timepoint	0.89 b	1.60 a	1.00 b	2.97 b	3.30 a	2.97 b	17.78 b	29.00 a	18.67 b

Different letters show statistically significant differences between treatments within each sampling point for p ≤ 0.05.

The two plant mixtures induced different effects on the soil microbiome. Mix1 did not affect microbial activity throughout the vegetative season, until the last sampling time, when it was reduced compared to control. However, the opposite pattern was observed for the biodiversity and richness indexes, which were both higher in spring time compared to control, but then returned similar to it in the following periods. Mix2 induced a different pattern dynamic for the bacterial activity compared to control, showing a lower activity in spring, higher in summer and similar in autumn. On the other hand, a positive steadily increasing pattern was observed for the other two indices, with lower values in spring, and higher in autumn than control (S2 Table).

**Fig 3:**
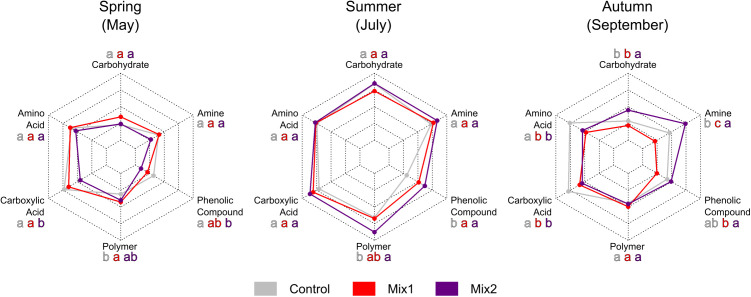
Comparison of the impact of two kinds of living mulch mixtures on bacterial activity (AWCD) towards certain C-sources classes during the vegetative season. Different letters show statistically significant differences between treatments within each C-source class and sampling point for p ≤ 0.05.

Irrespective of the different dynamic patterns observed for the three indices (AWCD, H and S), all classes of C-sources utilised by the bacterial community for its metabolism resulted to be significantly more exploited during summer (S2 Table and Fig 3). Compared to both spring and autumn (only for phenolic compounds the difference was not statistically significant). However, the two living mulches induced some modifications in the level of use of few classes of C-sources. In spring the metabolism of carboxylic acid and phenolic compounds was decreased by Mix2, while Mix1 induced an increase of the bacterial metabolic potential towards polymers, compared to control. In the summer, increased metabolism of polymers was induced by Mix2 and of phenolic compounds by both living mulches, compared to the control. Interestingly, the most significant changes in the metabolic potential of the soil microbiomes were observed in autumn: for some C-sources the effect of the two living mulches mixtures was similar (e.g., reduced metabolic potential towards amino acids and carboxylic acids), but for others divergent (increased metabolism of carbohydrates, phenolic compounds and amines by Mix2 but decreased by Mix1) (S2 Table and Fig 3).

**Fig 4:**
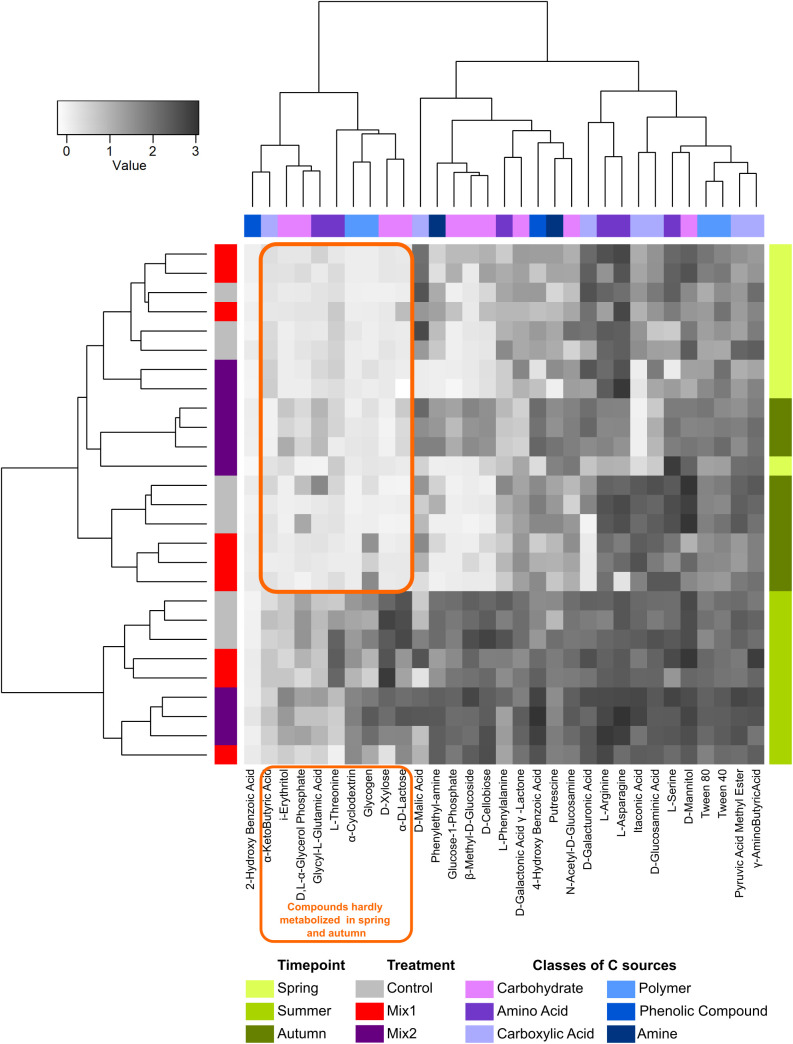
Microbial activity of the soil bacterial community towards various C-sources as affected by two kinds of living mulches and season.

Deepening the analysis of the impact of the two living mulch mixtures on the bacterial community C-source metabolism to individual compounds (Fig 4) allowed to distinguish a group of nine compounds that were hardly metabolized by microorganisms in spring and autumn belonging to four different classes of C-sources (carbohydrates, amino acids, carboxylic acids, polymers), but 4 out of 9 were among the carbohydrates. One compound (2-hydroxybenzoic acid) was barely used by microorganisms. On the other hand, several amino acids (e.g., L-arginine, L asparagine, etc.) and few organic acids (e.g., L-Malic acid) or polymers compounds (e.g., Tween) were instead commonly utilised by all bacterial communities of the three soil environments throughout the whole vegetative season (Fig 4).

**Fig 5:**
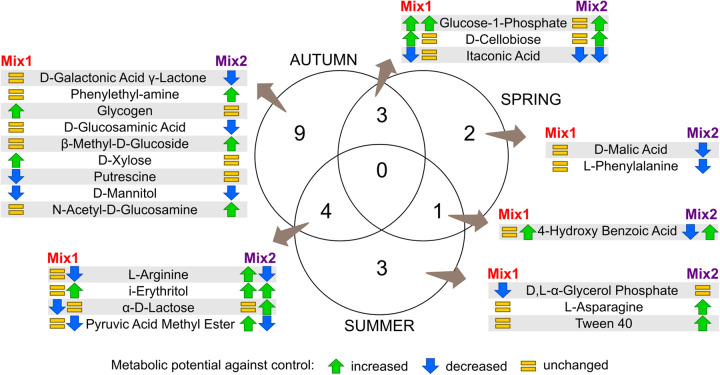
Venn’s Diagram visualizing significant changes in C-sources use according to changed bacterial activity between two living mulches mixtures when compared to the control, depending on the season.

The detailed analysis of differences in bacterial activity induced by the two living mulch mixtures within each sampling point showed that Mix2 induced a higher modification of the C-sources use by the bacterial microbiome compared to the control (23 out of 30) than Mix1 (14 out of 30), and the C-sources profiles were very contrasting (Fig 5, details in S3 Table). However, any C-source resulted to be differentially metabolised by both mixtures and control across the whole vegetative season.

Autumn was the period with the highest number of compounds with statistically significant changes (16 out 31 analysed C-sources), while in spring and summer a similar number of compounds resulted with changed activity (6 or 8 out of 31, respectively), though they were not the same C-sources, except for 4-hydroxy benzoic acid. On the other hand, a broader spectrum of compounds with significant changed activity was shared by the spring and summer samples with those of autumn (3 or 4 compounds respectively).

Intriguingly, all carbohydrates C-sources (n = 10) showed significant differences in the bacterial microbiome metabolic potential for at least one sampling point.

## Discussion

Both living mulch mixtures affected negatively, particularly during the first vegetative season, the initial growth of the apples trees compared to the control method of tree row management based on herbicide application and following natural cover. This effect impacted on the overall 2-year growth, particularly when considering the trunk diameter. Unexpectedly, such impact resulted more pronounced for Mix1, composed by a legume (*T. repens*) and a grass (*F. ovina*). The major rationale for testing this mixture was to combine a small growing grass with a N-fixing leguminous that would have reduced the competition for soil nutrients and provided nitrogen supporting the tree growth [33]. The soil nutrient level observed confirmed the correctness of the approach since the content of nitrate nitrogen was higher than in the control. However, this was not paralleled by the nitrogen content of the apple trees leaves, which was at the same level of the control. The association of *T. repens* with apple trees in a rhizobox system also resulted in trunk diameter growth reduction, but without negative effect on shoot growth (weight) and a higher root development [34].

In case of Mix2, which contained a lower proportion of *T. repens*, resulted in a limited negative impact on tree growth parameters, but still a N-leaf content similar to control and Mix1, though both N forms in the soil were higher than control only in summer. A possible explanation of this effect could be the presence of more plant species in Mix2, including *P. lanceolata*, which was reported to suppress soil N mineralization through exudation of various secondary metabolites interacting with the soil microbiome thereby reducing ammonia monooxygenase and nitrifiers activity [35,36]. This interaction may also explain the increase in ammonia levels and the almost no change of N-NO_3_ levels observed at different stages of the growing season in Mix2. A meta-analysis considering the impact of living mulches containing leguminous and grasses under various pedo-climatic conditions, showed they increased soil nutrients, but this was much less observed when the cover of the soil was only for a short term [37]. The negative effect of living mulches based on white clover on the tree growth after the orchard establishment was observed also under pedo-climatic conditions similar to those of the current study, but such negative impact was less evident when blue fescue was consociated with white clover or when a more vigorous rootstock was utilized [38]. However, the negative effects of this orchard floor management practice can be mitigated by delaying the introduction of living mulch for several years after the trees were planted [39,40].

The living mulch mixtures induced also a lower level of K in soil and in the apple tree leaves, which have likely affected tree growth and the yield potential [41,42]. However, it should also be considered that a persistent high soil nitrate nitrogen level could have an effect on the balance between vegetative and reproductive growth [43]. The fluctuations in soil Mg and Ca content did not always correspond to changes observed in the leaves Nevertheless, the changes of the macroelements levels in the leaves, even if statistically significant, still kept their content within the optimal or high content according to Polish standards for apple trees [44]. It is noteworthy that the evaluation of the living mulch mixtures impact considering all nutrients content allowed to discriminate between the two living mulches and the control, pointing to an effect that would comprehensively integrate the physiological role of each nutrient.

The introduction of mixtures of flowering plants in orchards (flower strips) is commonly considered a practice aiming at promoting functional agrobiodiversity [45], which generally is carried out in the alleyways or hedgerows of the orchard. In the present study we wanted to test the possibility of exploiting flower strips also for row management, which could also improve their expected performance on functional biodiversity [7]. Mix2, a living mulch mixture designed as a flower strip, had a limited negative impact on soil and tree nutrients status and tree growth compared to both control and the classical leguminous-grass living mulch (Mix1). Even though the performance of Mix2 on pest control or pollinators was not evaluated in the present study, the obtained results suggest that such approach could be a sustainable method for tree row management from the agronomical point of view, which could provide a variety of ecosystem services, without impairing the main crop performance.

The two living mulch mixtures generally modified soil bacterial activity and biodiversity and consequently the bacterial microbiome capacity of exploiting various growing substrates and C-sources. Such impact has been observed also in other studies on apple orchards [46,47]. Living mulches based only on white clover or a grass significantly modified various soil enzymatic activities (e.g., invertase, urease or alkaline phosphatase), and increased the soil bacterial carbon metabolic activity and the bacterial community diversity [48]. It is noteworthy that both living mulch mixtures induced significant fluctuations in the potential activity of soil bacterial population towards phenolic compounds compared to control. Both mixtures seemed to impact weed diversity (visual observations), and the proportion between monocotyledons and dicotyledons, similarly to other reports [49–52]. Considering that the concentration of phenolic acids in soil can range from 2.1% to 4.4% for the roots of monocotyledonous plants and from 0.1% to 0.6% for the roots of dicotyledonous plants [53], the different composition of the living mulches in this respect would account for the modifications of the bacterial population and its metabolic activity. Furthermore, the soil microbiome associated with another clover, *Trifolium pratense*, was found to include microorganisms belonging to the genera *Sphingobium* and *Novosphingobium* [54], which have a wide range of metabolic capabilities, including the degradation of phenolic compounds [55,56].

The soil microbiome associated with Mix1 showed a greater capacity to utilise glucose-1-phosphate and D-xylose in various periods of the vegetative season than the control, which may indicate the improved use of lignin or hemicelluloses derived from the living mulch plant residues as a carbon source [57]. Interestingly, the Mix2 soil bacterial microbiome showed similar properties only in autumn. The bacteria populations associated with the two living mulch mixtures differed in the activity towards various compounds that have been reported as plant root exudates (e.g., L-asparagine, L-serine, L-arginine, phenylethylamine, D-malic acid or 4-hydroxy benzoic acid, D-xylose, β-methyl-D-glucoside) of some species present in the mixtures, including clover [58–60]. Even though an unambiguous understanding of impact of the tested living mulches on the soil bacterial communities and their C-sources use requires more thorough research [61], it appears evident that the introduced living mulch mixtures, can significantly modify the activity and biodiversity of the soil microbiome in addition to the impact on above-ground biodiversity.

## Conclusions

In conclusion, the study demonstrated that a living mulch based on a mixture designed for establishing flower strips or to cover the soil in the interrow should not be sown into the apple tree rows during the orchard establishment, particularly when low vigour rootstocks (e.g., M9) are used, without additional interventions. Indeed, they competed for nutrient and water resources with the young apple trees, though they increased the soil N content and modified the bacterial community along the vegetative season. To limit the negative effects, the establishment of the living mulches could be complemented with either the application of organic or microbial biostimulants to the soil or foliar fertilization that can provide nutrients directly to the tree. Nevertheless, on a longer term, with the full development of the tree root system, the provision of organic matter and potential nitrogen fixation from both mixtures could partly balance the nutrient needs of the trees, particularly early season, allowing to reduce the needs of external inputs. This assumption is currently under evaluation as the limited time frame of the study can have potentially biased the observed overall effect of the living mulches. Nevertheless, the acquired knowledge could support the decision of farmers when implementing living mulches, are this practice is among those suitable for financial support within governmental programs meant to foster the adoption of cultivation methods enhancing biodiversity in orchards. Showing contrasting effects on trees for the two mixtures (which were selected also to provide additional ecosystem services) can thus provide scientific evidence for the practical implementation of the measure. The evaluation of the living mulches on yield and fruit quality, as well as on the containment of pests and weeds shall provide the conclusive information needed by operators. This assessment is currently ongoing.

It is noteworthy that the mixture commonly used to establish flower strips outside the orchard showed to be also suitable as a living mulch: its impact on beneficial fauna and reduction of apple pests in comparison to common flower strips is also currently under assessment.

The selection of any of these kinds of mixtures for a commercial orchard could thus depend on the kind of ecosystem service that the farmer considers more useful for its cropping system, whether it shall support crop protection or increase soil health. Future work should thus consider the identification of the best mixture according to the pedo-climatic conditions and the evaluation of the mixture’s impact on functional microbiome, which would shade light on the complex relationships established in the orchard soil.

## Supporting information

S1 Table Dynamic and concentration of soil nutrients and chemical parameters as affected by the introduction of two kinds of living mulches. Mean±SD, n = 3. Letters show statistically significant differences for p ≤ 0.05 (A-C for the same treatment in different timepoints, a-b for different treatments in the same timepoint).
(XLSX)

S2 Table Bacterial activity (AWCD) for six compound classes analysed with Biolog EcoPlate as affected by two kinds of living mulches mixtures during the vegetative season. Means±SD, n = 3. Different letters show statistically significant differences between sampling points within each compound class for p ≤ 0.05.
(XLSX)

S3 Table Bacterial activity between soil samples collected from tested treatments in different parts of the 2022 vegetative season. Different letters show statistically significant differences between treatments within each sampling point for p ≤ 0.05.
(XLSX)
